# How psychological empowerment impacts task performance: The mediation role of work engagement and moderating role of age

**DOI:** 10.3389/fpsyg.2022.889936

**Published:** 2022-09-16

**Authors:** Jesus Juyumaya

**Affiliations:** Millennium Nucleus on the Evolution of Work, Santiago, Chile

**Keywords:** psychological empowerment, work engagement, task performance, age, moderated mediation, human resources management

## Abstract

This paper presents a mediation–moderated model of the relationship between psychological empowerment, work engagement, age, and task performance. I seek to provide a more nuanced understanding of the mediating role of work engagement in the positive effect of psychological empowerment on task performance. Further, I explore employee age as a moderating factor in this mediation. I used online surveys among a sample of Latin American textile industry employees to capture individual perceptions about psychological empowerment, work engagement, and task performance. I modeled a mediation–moderated model using Hayes’ Process macro. The results confirm that the positive impact of employee psychological empowerment on task performance is partially mediated by work engagement. In addition, age was a significant moderator of the mediation effect. This study expands knowledge about how the psychological empowerment–work engagement relationship can predict task performance, including age as a boundary condition. Following the Job Demands–Resources theory, I also prove that conceptualizing psychological empowerment as a personal resource can benefit the integration of psychological empowerment and the work engagement stream of research. Moreover, the findings may help human resources management (HRM) researchers and practitioners acknowledge contextual differences in understanding the combined effects of psychological empowerment and work engagement. For instance, textile industry human resources managers can develop specific age–based human resource systems that empower and engages employees from emerging economies.

## Introduction

In organizational psychology and human resources management (HRM), there has been a growing interest in studying the antecedents of task performance. In specific, HRM in textile industry companies are primarily focused on task performance (International Labour Organization [ILO], 2021). The concept of task performance is defined as the employee’s behavior in pursuing the objectives set in advance. This performance is notably affected by the individual strategy to achieve these objectives (Maslach et al., 2001). This article expands current knowledge about how the psychological empowerment–work engagement relationship can predict task performance, including age as a boundary condition. Following the JD–R theory, I also prove that conceptualizing psychological empowerment as a personal resource can benefit the integration of psychological empowerment and work engagement theories.

Psychological empowerment represents the motivational construct of an intrinsic task, including four cognitions that reveal a personal orientation: competence, meaning, self–determination, and impact, and demonstrates cognitive directions about their job role (Spreitzer, 1995). Psychological empowerment and work engagement (Xanthopoulou et al., 2009; Bakker and Albrecht, 2018) have been related to individual positive results, such as task performance and wellbeing (Schaufeli and Bakker, 2003; Walumbwa et al., 2011). The job demands–resources (JD–R) theory is one of the most used theories to explain work engagement. Work engagement occurs when an employee has high job demands and resources to respond to these demands (Juyumaya, 2019). The JD–R model explains the employee’s motivational and strain process.

Resources are work–related elements that help face job demands (Demerouti et al., 2001). Resources can be of two types: (1) Personal resources if they refer to the individual’s self–perceptions of himself (e.g., self–esteem, self–efficacy, and optimism); and (2) Job resources if they are elements of the environment, physical, psychological, or organizational, which are available for the employee to face job demands (e.g., transformational leadership, autonomy, and feedback). The level of psychological empowerment of employees has been studied as an essential personal resource that increases the levels of work engagement (Zhang and Bartol, 2010). Nevertheless, researchers need to explore new mediators and moderators between these constructs and different types of job performance. Also, research needs to consider a wide range of samples (e.g., non–US samples, non–Students’ samples) and underexplored contexts (e.g., Latin America, textile industry). The study of work engagement moves away from the historical vision that has prevailed in the business world: the conception that a job is functional when the person performs their role and only dedicates themselves to it through a mechanized mode associated with the value chain. This is because work engagement theories study phenomena that had not previously been considered, such as vigor (energetic component), dedication (emotional component), and absorption (cognitive component) (Bakker and Demerouti, 2016).

This research explores the mediator role of work engagement in the relationship between psychological empowerment and task performance. Furthermore, I explore the role of employee age as a moderating factor in this relationship. In doing so, I seek to provide a more nuanced understanding of the mediating role of work engagement and the moderator role of age in the positive effect of psychological empowerment on task performance. Additionally, this research considers a poorly studied sample: Latin American textile industry employees. This is why this study diagnoses the levels of psychological empowerment, work engagement, and task performance of the employees in this industry, which is a valuable input for managerial decisions in HRM.

This article is structured as follows: first, this work develops the theoretical framework. Next, I explain the methodology. I modeled a first–stage mediation–moderated model using Hayes’ Process macro. The results confirm that the positive impact of psychological empowerment on task performance is mediated by work engagement. Interestingly, age was a significant moderator of the mediation effect. Finally, the final sections discuss the results, outline theoretical and practical implications for HRM, and present the conclusions of this study.

## Theoretical framework

### Psychological empowerment

Psychological empowerment is a phenomenon addressed by the field of organizational psychology. At the individual level, psychological empowerment is the ability of the person to feel responsible and the protagonist of their own life. At a corporate level, it is the opportunity for employees to be more efficient in their operation and take on creative challenges in their work and daily tasks (Spreitzer, 1995). The activation of individuals’ resources positively impacts the development of their functions at the individual, group, and organizational levels, allowing for sustained improvements (Deci et al., 1999; Schaufeli and Salanova, 2007).

According to Spreitzer (1995), psychological empowerment represents the motivational construct of an intrinsic task, including four cognitions that reveal a personal orientation: (1) Meaning, which refers to the alignment between one’s work role and one’s own beliefs, values, and standards; (2) Self–determination, is an individual’s sense of autonomy or control concerning the initiation or regulation of one’s actions; (3) Competence, refers to the belief in one’s capability to perform work activities successfully; and (4) Impact, is the belief that one can make a difference in the managerial process; that one could influence operational outcomes in the work unit. The four dimensions are independent, distinct, related, and mutually reinforcing, qualities that capture a dynamic state or active orientation toward work.

### Work engagement

Schaufeli and Bakker (2003) define work engagement as a positive, fulfilling, work–related state of mind characterized by vigor, dedication, and absorption. Following JD–R theory, work engagement is the mental state that occurs when employees have high job demands and increased job and personal resources to respond to these job demands. Job demands are the physical, psychological, social, or organizational aspects that require sustained physical and psychological effort and are associated with specific physiological and psychological costs (Demerouti et al., 2001). Job resources refer to the physical, psychological, social, or organizational aspects of the job that are functional in achieving work goals, reducing job demands and the associated physiological and psychological costs, or stimulating personal growth, learning, and development, while the personal resources if they refer to the individual’s self–perceptions of himself (Bakker and Demerouti, 2016).

Previous research (e.g., Xanthopoulou et al., 2009; Walumbwa et al., 2011; Salessi and Omar, 2016; Juyumaya, 2018) has shown that engaged employees will perform better than others. Work engagement should not be confused with concepts such as job satisfaction, organizational happiness, work addiction, and even its opposite, the mental state of exhaustion or sustained stress, known as burnout syndrome. Burnout is a feeling of failure and an exhausted and spent existence resulting from an overload due to the employee’s demands of energy, personal resources, or spiritual strength (Maslach and Leiter, 2008; Bernd and Beuren, 2021).

### Task performance

Task performance is another construct that has received the most attention from researchers of HRM, organizational behavior, strategic management, and organizational psychology. Possibly, it is famous since the competitiveness and productivity of organizations are closely linked to the individual performance of their members (Greguras et al., 2007). Hence, identifying its determinants and consequences has been a priority for various scholars, practitioners, and researchers. The lack of consensus about task performance measurement has led to the appearance of numerous scales to evaluate it. The specialized literature on task performance postulates more than eighty instruments to measure individual performance at a general level and more than forty scales to assess performance in more specific contexts (Wells and Welty–Peachey, 2011).

However, most of the instruments developed to date fail to measure all the dimensions that make up individual performance. On the other hand, using different scales to measure the dimensions of task performance can cause some redundancy in the questions, affecting the instrument’s application. It could even negatively impact the validity of the statistical analysis results due to increased correlations between the items (Murphy, 1990). Faced with the ambiguities caused by individual performance measurement, Koopmans et al. (2013) developed a generic instrument to evaluate it. The instrument, identified with the name of the personal task performance scale, has been designed to measure the behaviors of employees rather than their effectiveness.

The first attempts to explore generic task performance focused heavily on task requirements, with research focusing on technical competence, role performance, and task–specific competence, among others (Viswesvaran and Ones, 2000). Generic task performance models use broad dimensions to delimit the construct. However, models developed for specific jobs and contexts are based on narrower and personalized dimensions to describe the elements of task performance. Task performance is an essential dimension of generic task performance. It is found in the construct’s vast majority of explanatory models (e.g., Rodrigues and Rebelo, 2021).

Following Koopmans et al. (2013), task performance is composed of four dimensions: (1) Performance in the task; (2) Performance in context; (3) Counterproductive behaviors; and (4) Adaptive performance. These dimensions include the behaviors inherent to the technical tasks of the work position of the organization members. According to Koopmans et al. (2013), the performance of the task implies the performance of the specific or technical duties associated with the job. Therefore, it is related to the technical core of an organization and the activities directly or indirectly related to the transformation of organizational resources and capacities into appropriate products or services for economic exchange. This article analyzes the mediating effect of work engagement on the relationship between psychological empowerment and task performance.

### The age effect

The relationships between psychological empowerment, work engagement, and task performance can be affected by demographic elements linked to individuals’ attitudes, values, and behaviors (Hofstede et al., 2010). Workforce aging and the need to work longer imply several challenges worldwide (Yeves et al., 2019). This paper proposes that the evolution of society, expressed in the emergence of different generational cohorts (e.g., baby boomers and centennials), might affect how employees experience psychological empowerment. For instance, the twenty-first century brought more individualism, competitiveness, and pressure to succeed. It increased the expectations of more horizontal relations and reduced power distance. In this context, the need for recognition was enhanced among younger employees, given the desire to excel among peers and establish closer relations with the leader (Didier and Luna, 2017). Then, I suggest that psychological empowerment on task performance will be more assertive in younger generations vs. older generations (baby boomers and generation X vs. millennials and centennials).

Previous research on social exchange theory has shown that reciprocity norms are more important among shortly tenured employees (Bal et al., 2013). As individuals shape their perceptions of the nature and dynamics of an exchange relationship, the reception of social benefits by the employer can boost a more favorable exchange expectation in younger employees. Similarly, as younger generations begin to understand and make sense of employment relationships through their interactions with organizational agents, psychological empowerment should differentially create more positive changes in them, in contrast to more experienced employees who have developed a more classical mindset of their employment relationship. In Latin America, this moderating effect could be more prevalent, as collectivism is inherently rooted in creating positive social exchanges based on reciprocity.

**FIGURE 1:**
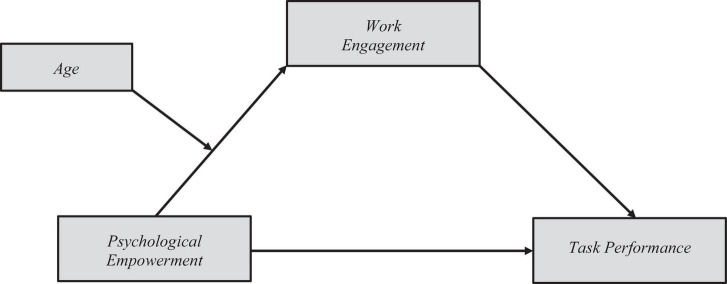
Proposed model.

These collectivistic values could be learned by employees before their initial working experiences but are reinforced and confirmed when interacting with leaders. Moreover, Atwater et al. (2009) suggested that feedback is less likely to be found from supervisors in cultures such as Latin American culture. It is easier to obtain feedback from peers. Therefore, it is more presumable that the effects of psychological empowerment are more meaningful in younger generations. On this basis, I propose a mediation–moderated model. Figure 1 shows the model and the following hypothesis,

Hypothesis: The mediating role of work engagement in the relationship between psychological empowerment and task performance is moderated by employee age, with the effect being more substantial for younger employees.

## Methods

### Data collection and sample

I designed a cross–sectional study and followed a quantitative approach using surveys. The online survey method was used for data collection. This method has multiple benefits, like a higher response rate compared to the manual distribution of a questionnaire (Rasool et al., 2021). I used Google Forms to conduct the experiment and automatically collected all the responses. This will allow the validation of the hypothesis through statistical analysis.

The study population was textile industry employees from Chile. The population was 655.257 employees in 2019 (SOL Foundation, 2022). Sample size = 196 (95% confidence). Employees worked in different communes of the metropolitan region of Chile. The final sample consisted of 200 employees (*n* = 200; female = 80%). I note that “The Chilean Social Outbreak” and the “Coronavirus Pandemic” did not affect the process because the data collection was carried out between January and September 2019.

### Measures

The methodology used is supported by a positivist epistemology, which promotes knowledge based on the formulation of hypotheses and empirical verification through the scientific method. This methodology requires data collection instruments that maintain the objective nature of the research. To this end, the present study used a survey that contained three Likert–type scales: (1) Psychological empowerment; (2) Work engagement; and (3) Task performance. Table 1 shows the items and authors of the used scales.

**TABLE 1:** Measures.

Construct	Items	References
Psychological empowerment (PE)	PE1. I am confident about my ability to do my job.	Spreitzer, 1995
	PE2. The work that I do is important to me.	
	PE3. I have significant autonomy in determining how I do my job.	
	PE4. My impact on what happens in my department is large.	
	PE5. My job activities are personally meaningful to me.	
	PE6. I have a great deal of control over what happens in my department.	
	PE7. I can decide on my own how to go about doing my own work.	
	PE8. I have considerable opportunity for independence and freedom in how I do my job.	
	PE9. I have mastered the skills necessary for my job.	
	PE10. The work I do is meaningful to me.	
	PE11. I have significant influence over what happens in my department.	
	PE12. I am self–assured about my capabilities to perform my work activities.	
Work engagement (WE)	WE1. At my work, I feel bursting with energy.	Schaufeli et al., 2006
	WE2. At my job, I feel strong and vigorous.	
	WE3. When I get up in the morning, I feel like going to work.	
	WE4. I am enthusiastic about my job.	
	WE5. My job inspires me.	
	WE6. I am proud of the work that I do.	
	WE7. I feel happy when I am working intensely.	
	WE8. I am immersed in my work.	
	WE9. I get carried away when I’m working.	
Task performance (TP)	TP1. How do you rate the quality of your own work in the past 3 months?	Koopmans et al., 2013
	TP2. Compared to last year, I judge the quality of my work in the past 3 months to be.	
	TP3. How often was the quality of your work below what it should have been in the past 3 months?	
	TP4. How do you rate the quantity of your own work in the past 3 months?	
	TP5. Compared to last year, I judge the quantity of my work in the last 3 months to be.	
	TP6. How often was the quantity of your work less than it should have been in the past 3 months?	
	TP7. I managed to plan my work so that it was done on time.	
	TP8. I worked toward the end result of my work.	
	TP9. I kept in mind the results that I had to achieve in my work.	
	TP10. I had trouble setting priorities in my work. TP11. I was able to separate main issues from side issues at work. TP12. I was able to perform my work well with minimal time and effort. TP13. It took me longer to complete my work tasks than intended.	

The applied survey consisted of a presentation of the study, the informed consent that ensured the confidentiality of the data and anonymity of the respondent, socio–demographic questions, and the three scales previously mentioned. All the scales followed a 5–point Likert–type response format, from “Strongly Disagree” = 1 to “Strongly Agree” = 5.

#### Psychological empowerment

Empowerment scale (Spreitzer, 1995). Spreitzer’s psychological empowerment scale (1995) measures perceived control, perceptions of competencies, and internalization of organizational goals and objectives. This research uses the 12 items Spanish validated version of Rivera et al. (2020).

#### Work engagement

UWES–9 scale (Schaufeli et al., 2006). This scale is available in two versions, including 17 items, and the abbreviated version, which includes nine items. I use the abbreviated version validated in Spanish by Juyumaya (2019).

#### Task performance

The Koopmans et al. (2013) task performance scale was used. The 13 items scale validated in Spanish by Gabini and Calzada (2015) was used.

#### Age

The study asked the age of each participant at the beginning of the survey. Then, I split the sample into two groups (high age/low age) to create a dummy variable.

#### Controls

In line with previous research (Walumbwa and Hartnell, 2011), we included participants’ age, gender, and work experience as control variables, because these variables can influence task performance.

### Analysis strategy

The analyses were carried out using the statistical package SPSS v.23 and the Process macro extension (Hayes, 2013). The analysis strategy aimed to corroborate the existence of a possible mediation–moderated between the variables selected for the study. The data interpretation of this research is based on model 7 proposed by Hayes (2013). The normality test used was Pearson’s Chi–square, which allowed us to justify a parametric study. No missing data was found in this survey.

## Results

Descriptive statistics, correlations, and Cronbach’s alpha are available in Table 2. The correlations are positive and align with what has been reported. In the three variables used, the alpha exceeds the value of 0.80, which indicates that the scales were reliable.

**TABLE 2:** Descriptive statistics and correlations.

Variables	M	SD	Minimum	Maximum	1	2	3
1. Psychological empowerment	3.69	0.77	1.00	5.00	**(0.88)**		
2. Work engagement	4,12	0.70	1.00	5.00	0.36**	**(0.98)**	
3. Task performance	3,43	0.64	1.00	5.00	0.22**	0.81**	**(0.89)**
4. Age	45.62	13.52	18	86	0.34**	0.38**	0.51**

Alpha scores on parentheses and bold. **p < 0.01.

### Confirmatory factor analysis

This research conducted a confirmatory factor analysis (CFA). I tested the hypothesized three–factor model with psychological empowerment, work engagement, and task performance. The model fitted well the data [x2(108) = 2330.21; CFI = 0.98; SRMR = 0.05; RMSEA = 0.05], suggesting that participants were able to distinguish our key constructs. I also ran four alternative models merging pairs of constructs (psychological empowerment–work engagement; psychological empowerment–task performance; work engagement–task performance) and one model with a single–factor solution. Neither of these alternative models showed better fit indexes than the hypothesized model.

### Mediation–moderated analysis

The main results of the mediation–moderated analysis are presented in Table 3. Table 3 shows the estimated and bootstrapped internals of work engagement (mediator) in the relationship between psychological empowerment and task performance for different levels of age (moderator) (+1 SD, M, −1 SD). I observe that the estimate of the mediation effect is significant and relatively constant at different levels of the moderator. The confidence interval of moderated mediation was different from zero [Estimate = 0.16, BootSE = 0.08, 95% CI: (0.19; 0.40)], suggesting that the mediation effect was moderated by employees’ age (Hayes, 2013).

**TABLE 3:** Indirect effects at Age = M ± 1 SD.

Indirect effect	Estimate	LLCI 95%	ULCI 95%
–1.00 SD	0.85	0.019	0.145
0.00 SD	0.16	0.190	0.401
1.00 SD	0.18	0.093	0.024

The table shows the bootstrapping procedure results using Hayes’s Process macro to test conditional effects (–1 SD, M, and +1 SD) for the moderator variable (Age). LLCI, Lower level 95% confidence interval; ULCI, Upper level 95% confidence interval. A bootstrapping procedure used 5,000 random subsamples to produce a normal distribution.

**FIGURE 2:**
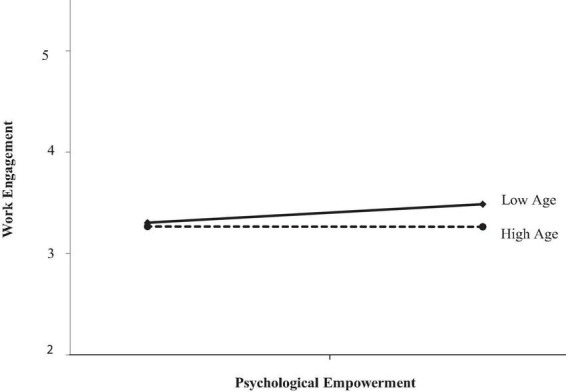
The moderating effect of age.

In Figure 2, the slope representing the relationship between psychological empowerment and work engagement is positive for young employees and different from the same slope for older employees. Younger employees (low age: SD = −1.00) have higher engagement levels as perceived psychological empowerment increases. In comparison, work engagement levels for older employees (high age: SD = 1.00) appear unaffected by the psychological empowerment effect supporting the study’s hypothesis.

## Discussion

Thanks to the results presented, the hypothesis of this study was supported. It is concluded that if employees are empowered in their capacities (i.e., with high levels of psychological empowerment), they will perform better when fulfilling their tasks. Furthermore, suppose employees are engaged (i.e., with high levels of work engagement). In that case, they will have even more positive repercussions on task performance. Moreover, I found that employees’ age moderates the mediation of psychological empowerment, work engagement, and task performance such that the mediation effect is more substantial for younger employees.

At the level of theoretical contribution, this study contributes to the theory of JD–R, which explains work engagement (Bakker, 2018). Psychological empowerment is a personal resource that helps respond to employees’ job demands in the textile industry. Hence, this study provides empirical evidence to understand work engagement in emerging economies. Scholars engaging in aging and HRM must study how different generational cohorts of employees experience psychological empowerment. Following other studies (e.g., Yeves et al., 2019), this paper delivers empirical evidence that the emergence of different generational cohorts (e.g., baby boomers and centennials) might affect how employees experience psychological empowerment and work engagement, and task performance.

The results support that work engagement mediates the relationship between psychological empowerment and task performance. Psychological empowerment is a crucial resource for facing job demands. Engaged employees have the energy (i.e., vigor), positive feelings (i.e., dedication), and attention (i.e., absorption) to perform better in the tasks (Bakker and Demerouti, 2016). As I mention, task performance is crucial for all industries, but even more in the textile industry. The presented results support the idea of Schaufeli and Salanova (2007) that engaged employees have more efficacy in their daily tasks. Engaged employees are happier at work, and their wellbeing and positive psychological state impact their task performance.

### Practical implications

An essential significance of this study is related to the possibilities that the HRM in the textile industry has to create opportunities for employees to increase their levels of psychological empowerment and work engagement. These results highlight the prominence of recognizing the capabilities and attributes of the employees of an organization because if you are outstanding, the employees acquire security far beyond the work, reaching a personal level, which can bring multiple benefits to the individual, even beyond the work dimension (Rappaport, 1981; Riger, 1993). For instance, 360° performance evaluations would be quite indicated in the textile industry. This type of evaluation encourages the employee to empower himself and improve their task performance based on feedback from his co-employees, supervisors, customers, or clients and their own self–assessment.

This study delivers empirical evidence to scholars interesting in the textile industry. Scientific papers focused on employees in the textile industry are scarce. For this reason, gathering factual data regarding employees in this industry is a methodologically relevant task. The textile industry has similar features concerning the workforce composition in all the world countries since it mainly comprises women (Fashionunited, 2020). In the present research, the percentage of female participants was 80%. I encourage future studies to analyze the role of gender in the relationship between HRM practices and task performance or other contingent relationships.

The findings of this study can help business and management researchers and practitioners acknowledge contextual differences in understanding the combined effects of psychological empowerment and work engagement. Managers and practitioners may develop a specific age–based HRM system that empowers and engages employees. For example, the individually–driven work design process (i.e., job crafting) can better align the job with personal needs, goals, and skills (Wijngaards et al., 2022). Embedding strategies in people management practices that promote psychological empowerment and work engagement is a crucial source of competitive advantage based on developing individual capacities that are difficult to imitate. For instance, HRM areas can create organizational innovation strategies. These actions can build a positive corporate culture that benefits psychological empowerment and work engagement through supportive generational–based feedback (e.g., millennials mentoring baby boomers, and vice–versa) and, at the same time, influence sustainable organizational performance (Rasool et al., 2019).

### Limitations and future research

Further research can study aspects related to the limitations of the present study. One limitation is the risk of common method variance due to using self–reported data. Future research can make an effort to solve this. Other limitations concern the study’s sample. Scholars from a wide range of perspectives should study employees in the textile industry from other Latin American countries, conduct longitudinal studies, and conduct comparative analyses between culturally different countries. Additionally, future research could continue to delve into other job or personal resources that increase work engagement (Juyumaya and Torres, 2020). For example, the study of the impact of new technologies and work arrangements on task performance, focusing on textile industry employees, can be a source of exciting future directions.

## Conclusion

This paper presents a mediation–moderated model that analyzes the role of work engagement as a mediator in the relationship between psychological empowerment and task performance in the textile industry. In this way, I postulate that psychological empowerment increases work engagement, which in turn has, as a consequence, a higher task performance of the employees of this industry. Additionally, an employee’s age moderates the mediation effect of work engagement in the relationship between psychological empowerment and task performance, such that the mediation effect is more substantial for younger employees.

This article provides empirical evidence to develop the psychological empowerment, work engagement, and aging literature. Moreover, because this study investigates a poorly studied sample, it provides inputs to further theoretical analysis. For managerial practice, this study helps to manage organizations based on evidence. I promote that HRM managers consider psychological empowerment and work engagement as essential employee results. Likewise, the generation factor must be addressed. Thus, companies and businesses can promote the quality of working life, improve task performance, and build thriver organizations.

## Data availability statement

The raw data supporting the conclusions of this article will be made available by the authors, without undue reservation. Requests to access the datasets should be directed to the corresponding author.

## Ethics statement

The studies involving human participants were reviewed and approved by the Universidad Santo Tomas. The patients/participants provided their written informed consent to participate in this study.

## Author contributions

The author confirms being the sole contributor of this work and has approved it for publication.
